# Retraction: Choosing the most appropriate minimally invasive approach to treat gynecologic cancers in the context of an enhanced recovery program: Insights from a comprehensive cancer center

**DOI:** 10.1371/journal.pone.0338982

**Published:** 2025-12-18

**Authors:** The PLOS One Editors

The *PLOS One* Editors retract this article [1] because it was identified as one of a series of submissions for which we have concerns about compromised peer review.

AN and EL did not agree with the retraction. CJ, CB, LS, GB, and GH either did not respond directly or could not be reached.
